# Phylogenetic characterisation, virulence factors, and multi-drug resistance of *Escherichia coli* strains isolated from faeces of feral pigeons (*Columba Livia* forma *urbana*)

**DOI:** 10.1186/s13620-025-00306-2

**Published:** 2025-08-25

**Authors:** Katarzyna Kowalczyk, Angelina Wójcik-Fatla

**Affiliations:** Department of Health Biohazards and Parasitology, Institute of Rural Health, Jaczewskiego 2, Lublin, 20-090 Poland

**Keywords:** *Escherichia coli*, Virulence, Drug resistance, Urban environment, Public health, Pigeon

## Abstract

**Background:**

Feral pigeons are a synanthropic species commonly found in cities worldwide. They are known to carry zoonotic pathogens, including *Escherichia coli*, and have long raised concerns about environmental contamination and public health risks.

**Objectives:**

The aim of the study was to phylogenetically classify, identify selected virulence genes and determine the phenotypic antimicrobial susceptibility profiles of *E. coli* isolated from pigeon faeces in urban agglomeration.

**Methodology:**

A total of 120 fresh faecal samples were collected from feral pigeons in urban areas. Groups of 4 samples from each location were tested in a total of 30 pools. A total of 97 faecal *E. coli* isolates were screened for enteropathogenic *E. coli* (EPEC) and Shiga toxin-producing *E. coli* (STEC) strain genes and thirteen selected virulence factors associated with pathogenic function and activity. Resistance patterns were determined by the Kirby-Bauer disk diffusion method for twenty antibiotics.

**Result:**

The most common phylogenetic group was group D (70/97, 72.2%), followed by group A (15/97, 15.5%), B1 (7/97, 7.2%) and B2 (3/97, 3.1%). EPEC and STEC were found in 5.2% and 22.7% isolates, respectively. The obtained results showed *katP*, *lpfA*_*O157/OI−141*_, *tir*, *iha* and *lpfA*_*O157/OI−154*_ genes in *eaeA*-positive and *stx*-positive isolates, mainly from phylogroups D and B2. The isolated *E. coli* strains were resistant to at least one antibiotic in 16.5%, and 2.1% were recognised as multidrug-resistant (MDR).

**Conclusions:**

The results of this study confirm that pigeons in the urban environment are carriers of potentially pathogenic strains of *E. coli*, including MDR strains. Twelve patterns of virulence genes were identified among *E. coli* strains, with a great predominance of the single gene *stx*_*1*_ encoding Shiga toxin 1. The highest resistance was observed for imipenem (IMP), tetracycline (TE) and doxycycline (DO), respectively, and these antibiotics were also involved in most of the observed resistance patterns. The obtained results justify the implementation of preventive measures in cities and the introduction of surveillance programs for synanthropic pigeon populations to protect both the urban environment and public health.

**Supplementary Information:**

The online version contains supplementary material available at 10.1186/s13620-025-00306-2.

## Introduction

*Escherichia coli*, gram-negative bacteria belonging to the *Enterobacteriaceae* family, is one of the most diverse and genetically variable microorganisms, divided into at least 11 different pathotypes in two main categories: intestinal pathogenic *E. coli* (InPEC/IPEC) and extraintestinal pathogenic *E. coli* (ExPEC) [1].

Pathogenic strains of *E. coli* can cause many forms of infections, along with a wide range of symptoms, for example in the digestive system, circulatory system, respiratory system and urinary tract. In some cases, these infections threaten human health and life [1, 2]. *E. coli* is one of the main causes of bacteraemia leading to sepsis and has a mortality rate of approx. 15% [3, 4].

Human infections most often occur through consumption of raw and undercooked food (meat, meat products, fruits, vegetables, dairy products) or contaminated drinking water [5, 6]. The bacteria can enter the environment (e.g., water and soil) via the faeces of wild animals and migratory birds [7, 8], but also as a result of improper sewage and livestock manure management [9–11]. Non-compliance with hand hygiene protocols may also lead to bacteriological contamination of environmental surfaces [12, 13].

Despite the large genetic and phenotypic diversity of pathogenic *E. coli* strains, the species has a clonal population structure [14], consisting of at least eight major phylogenetic groups (A, B1, B2, D, E, F, G and H) [4]. It has been shown that pathogenic *E. coli* isolates from different human and animal hosts and environments can share a common genetic background, but may differ in pathotypes and phylogenetic structure [1, 4, 14]. The pathogenicity of *E. coli* pathotypes depends on their virulence factors, which determine the mechanisms of adaptability to colonise various niches in host organisms. There are many genetic determinants of virulence that can be grouped into several classes depending on activity or function. They include virulence factors associated with extraintestinal and intraintestinal infections, related to colonisation (adherence, attachment, biofilm formation, adhesion fimbria), fitness, toxin production (Shiga toxins, heat-stable enterotoxins), and effectors (effector proteins, translocator proteins of type III secretion system (T3SS) [14, 15].

The presence of one or several specific virulence factors determines the development of the disease. Enterohemorrhagic *E. coli* (EHEC) serotype O157:H7 may cause severe diseases with potentially devastating or life-threatening complications of gastroenteritis, haemorrhagic colitis, and haemolytic uremic syndrome [16]. Recently, attention has been paid to increasing reports of outbreak and non-outbreak infections caused by other serotypes and hybrid strains of *E. coli* with multi-pathovar features [17, 18], such as the rare hybrid of enteroaggregative *E. coli* and STEC O104:H4 [19].

Wild birds, including feral pigeon populations in urban areas, are carriers of pathogenic *E. coli*, such as STEC, EPEC, EHEC strains, and MDR strains [20, 21]. Due to high genotypic and phenotypic plasticity, these bacteria can survive in urban environments, where they acquire and transfer virulence and resistance genes through various mechanisms, e.g., mobile genetic elements, mutations. Exposure to environmental factors, such as urban dust contaminated with faecal particles containing the pathogenic *E. coli*, poses potential risk to human and animal health [22] and favours the transition of commensal strains into more pathogenic variants [23, 24]. Moreover, selection pressure provided by the environment may be more important for the acquisition of adaptive and virulent traits and for increasing antimicrobial resistance in *E. coli* populations than genetic variability resulting from mutations [25, 26]. *E. coli* is one of the priority species in terms the global problem of antibiotic resistance [27], therefore, extensive monitoring, covering both clinical and environmental strains, is required to implement preventive strategies [28, 29].

The aim of the study was the phylogenetic classification, identification of selected virulence genes and determination of phenotypic profiles of antimicrobial susceptibility of *E. coli* strains isolated from fresh faeces of feral pigeons with regard to human exposure to zoonotic hazards in urban areas.

## Materials and methods

### Sample collection

A total of 120 fresh faecal samples from feral pigeons (*Columba livia* forma *urbana*) were collected from April to September 2022 in the city of Lublin, Poland. Samples were collected from 30 locations in the area of approx. 18.5 ha, in public places characterised by a high density of urban pigeons (groups of more than 15 birds) and observed environmental contamination with bird faeces: in parks (5 locations), city squares (6 locations), residential areas (14 locations), and near hospitals (5 locations). To reduce the risk of cross-contamination from the ground, only fresh and single faecal samples were collected using a sterile wooden disposable ENT (Ears, Nose, and Throat) spatula and placed into sterile tubes. The pooled sample consisted of 4 individual faecal samples collected at a determined location; a total of 30 pools were tested. The pooled samples were mixed thoroughly in a Petri dish using a sterile spatula for homogenisation.

### Microbiological culture

**Fig. 1:**
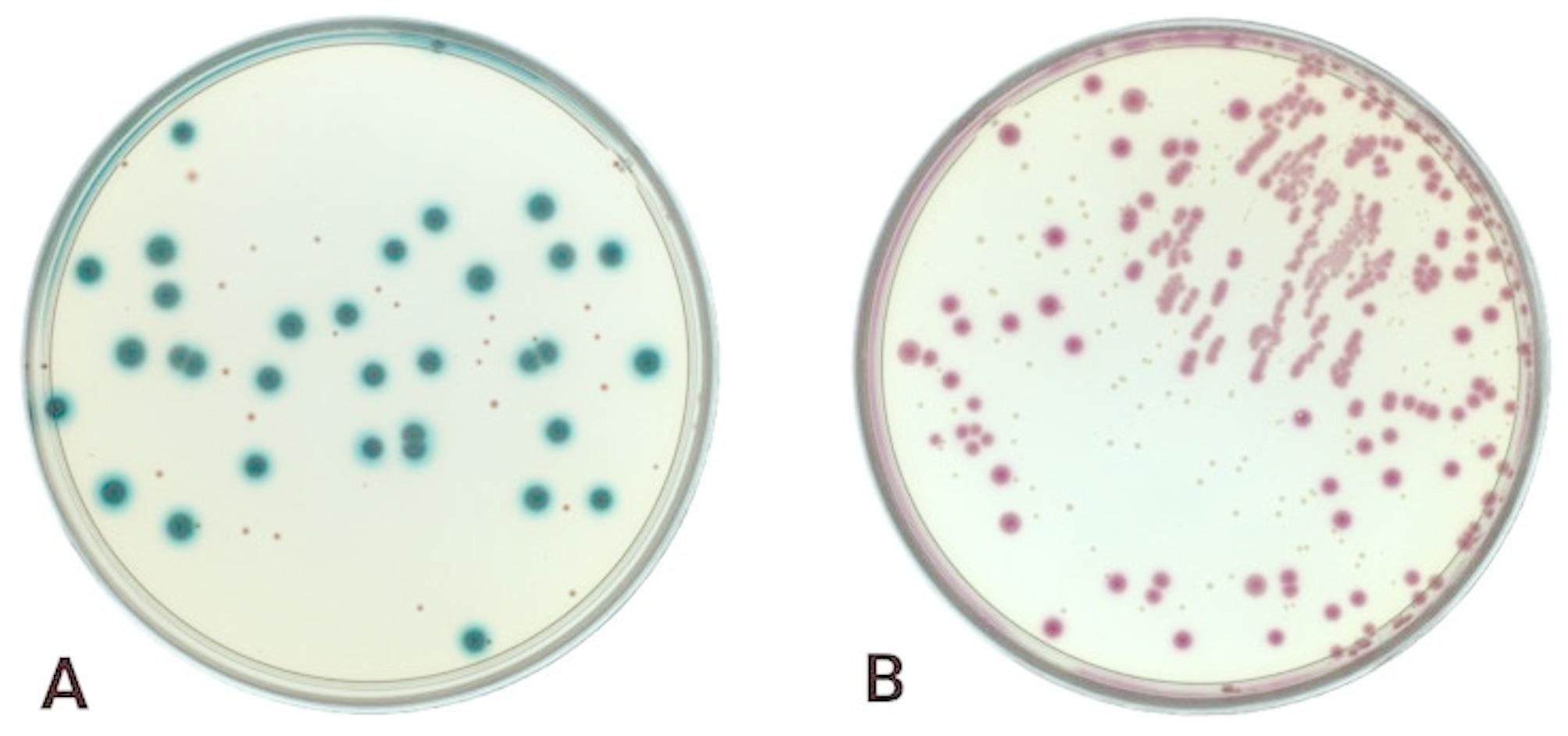
Microbiological cultures of *E. coli* isolated from urban pigeon faeces samples on R + EB medium (**A**) and CC medium (**B**)

A 1.0 g of homogenised sample was taken from each of the 30 pools, and a series of 10-fold dilutions were made in Ringer’s solution (10^−1^ – 10^−6^). Microbiological cultures were surface inoculated on two selective media: Rebecca agar with EB Supplement (R + EB) (bioMérieux, Marcy-l’Étoile, France) and ChromID Coli (CC) agar (bioMérieux, Marcy-l’Étoile, France). The plates were incubated aerobically at 37 ± 1° C for 18–24 h. Based on the morphological characteristics, typical blue β-D-glucuronidase-positive *E. coli* colonies with or without halo were counted only from R + EB medium (Fig. 1). Results were expressed as colony-forming unit per gram (CFU/g). Phenotypically suspect *E. coli* colonies from R + EB and CC were subcultured in nutrient agar (BTL, Łódź, Poland) and incubated at 30° C for 24 h for further biochemical testing, DNA extraction and molecular testing.

### Biochemical tests

Identification of *E. coli* strains was carried out using ENTEROtest 24 N (Erba-Lachema, Brno, Czech Republic) according to the manufacturer’s recommendations. The result was received using a semi-automatic ErbaScan reader (Erba-Lachema, Brno, Czech Republic) and the ErbaExpert microbiological software (Erba-Lachema, Brno, Czech Republic).

### Antimicrobial susceptibility testing

Antimicrobial susceptibility testing was performed using the Kirby-Bauer disc diffusion method on Mueller-Hinton (MH) agar plates (Biomaxima, Lublin, Poland). Twenty antimicrobials from 9 different antibiotic classes were testing: ampicillin (AM; 10 µg), ampicillin-sulbactam (SAM; 10 and 10 µg), amoxicillin-clavulanic acid (AMC; 20 and 10 µg); azithromycin (AZM; 15 µg); aztreonam (ATM; 30 µg); ceftazidime (CAZ; 30 µg); cefuroxime (CXM; 30 µg); chloramphenicol (C; 30 µg); doxycycline (DO; 30 µg); imipenem (IMP; 10 µg); kanamycin (K; 30 µg); meropenem (MEM; 10 µg); nalidixic acid (NA; 30 µg); ofloxacin (OFX; 5 µg); piperacillin (PRL; 100 µg); piperacillin-tazobactam (TPZ; 100 and 10 µg); streptomycin (S; 10 µg); tetracycline (TE; 30 µg); ticarcillin-clavulanic acid (TTC; 75 and 10 µg); trimethoprim-sulfamethoxazole (TS; 30 µg).

A standardised bacterial suspension with an optical density of 0.5 McFarland standard was spread on MH media. Antibiotic discs (Biomaxima, Lublin, Poland) were placed on inoculated plates and incubated at 35 °C for 18 h. *Escherichia coli* strain ATCC 25,922 was used as the quality control. The breakpoint of zone diameters (measured in mm) was interpreted in accordance with the recommendations of the European Committee on Antimicrobial Susceptibility Testing (EUCAST) [30] and the Clinical and Laboratory Standards Institute (CLSI) [31].

### Molecular identification

The genomic DNA of *E. coli* strains was isolated using the QIAamp DNA Mini Kit (Qiagen, Hilden, Germany), according to the manufacturer’s instruction with 100 µl final volume of DNA elution. Bacterial DNA was detected by amplification of the 16 S rRNA fragment genes using primers: p27f_F/p1525r_R [32], and 16E1/16E2, 16E1/16E3 [33] to differentiate “*pathogenic*” and “*non-pathogenic*” *E. coli* strain, respectively. PCR reactions were performed using C1000 Thermal Cycler (BioRad, Hercules, CA, USA) and Mastercycler^®^nexus (Eppendorf, Hamburg, Germany). Amplification products were identified in 1.5% or 2% agarose gel and visualized using a gel documentation and image analysis system (InGenius LhR, Syngene, UK) after 2 µg/ml ethidium bromide staining.

Sequencing was performed with ABI PRISM 310 Genetic Analyzer (Applied Biosystems, Waltham, MA, USA) using ABI PRISM Big Dye Terminator v. 3.1. Cycle Sequencing Kits and Big Dye XTerminator Purification Kit (Applied Biosystems, Waltham, MA, USA). The results of the nucleotide sequences were compared with data stored in GenBank database using the Basic Local Alignment Search Tool software at the National Centre for Biotechnology Information (Bethesda, MD, USA). The sequences obtained in the current study were deposited in GenBank database.

Components and conditions of all PCR reactions performed in this study were listed in Tables S1 and S2 (Supplementary Material).

### Phylogenetic group determination

Identification of the four main basic phylogenetic groups (A, B1, B2, D) was determined in a triplex PCR reaction according to the procedure described by Clermont et al. [34] (Table S1, S2). The phylogenetic group was determined based on a dichotomous decision tree: *chuA* and *yjaA* positive (group B2), *chuA* positive and *yjaA* negative (group D), *chuA* negative and TSPE4.C2 positive (group B1), *chuA* and TspE4.C2 negative (group A).

### Virulence genes detection

The detection of *stx*_*1*_ and *stx*_*2*_ (Shiga toxin 1 and 2), *eaeA* (intimin) and *hlyA* (enterohemolysin A) genes was determined by multiplex PCR according to Kim et al. [35] (Table S2). The isolates were also tested for thirteen selected virulence genes associated with colonisation and effectors, including: *tir* (translocated intimin receptor), *espA* (translocator protein of T3SS), *espB* (translocator protein of T3SS), *katP* (catalase-peroxidase enzyme), *espP* (extracellular serine protease), *etpD* (protein D of type II secretion pathway), *saa*, *sab* and *toxB* (biofilm formation and adhesion), *iha* (IrgA homologue adhesin), *lpfA*_O157/OI−141_, *lpfA*_O113,_ and *lpfA*_O157/OI−154_ (long polar fimbriae) genes. The PCR reactions were performed by multiplex PCR according to Monaghan et al. [36]. *Escherichia coli* strain O157:H7 ATCC 43,890 was used as a positive control.

### Detection of efflux pump encoding genes

Three genes *acrA*, *acrB*, *tolC* encoding components of AcrAB-TolC efflux pump were assayed by monoplex PCR reactions targeting the membrane fusion protein acrA, inter membrane protein acrB and outer membrane factor TolC, according to the methodology described by Olubisose et al. [37] (Table S1, S2).

## Results

### Presence of *E. coli* strains in pigeon faecal samples

The presence of *E. coli* was found in each of the 30 sample pools tested. Considering the total number of faecal samples (120), the minimum infection rate was 25% [38] whereas the maximum likelihood estimation was 100% [39].

The average total number of β-D-glucuronidase-positive *E. coli* from R + EB plates was 8.78 × 10^7^ CFU/g ranging from 1.70 × 10^5^ to 4.75 × 10^8^ CFU/g. A total of 100 isolated bacteria strains were classified as *E. coli* species using selective microbiological culture method and biochemical testing.

### Phenotypic antibiotic resistance of *E. coli* strains and efflux pump

Of all *E. coli* strains tested, 16/97 (16.5%) were resistant to at least one antimicrobial agent tested, and 2/97 (2.1%) were found to be MDR with resistance to three or more classes of antibiotics. The most frequently observed resistance was imipenem (9/97, 9.3%) of carbapenems class, tetracycline (7/97, 7.2%) and doxycycline (5/97, 5.2%) of tetracyclines class (Table 1). All tetracycline-resistant strains were simultaneously resistant or intermediate sensitive to doxycycline (5/7, 71.4% and 2/7, 28.6%). The lowest resistance was found to amoxicillin-clavulanic acid (3/97, 3.1%) and piperacillin (1/97, 1.0%) of penicillins class, ofloxacin (3/97, 3.1%) of fluoroquinolones class, and aztreonam (2/97, 2.1%) of monobactams class. All *E. coli* strains were intermediate sensitive to cefuroxime for cephalosporins class. A total of ten resistance patterns were observed for individual antibiotics (Table 2). None of the *E. coli* strains showed resistance and intermediate sensitive to antibiotics from miscellaneous agents, macrolides, lincosamides, and streptogramins class (Table 1).

**Table 1:** Sensitivity profiles of *E. coli* strains to antimicrobial agents of different antibiotic classes

Class of antibiotics	Antibiotic	Average of zone of inhibition (range) [mm]	Results of susceptibility* (No.**) *n* = 97			
			*R*	I	S	
Penicillins	ampicillin (AM)	20.8 (17–29)	0	0	97	
	ampicillin-sulbactam (SAM)	23.6 (20–34)	0	0	97	
	amoxicillin-clavulanic acid (AMC)	22.4 (18–26)	3	0	94	
	piperacillin (PRL)	28.4 (17–33)	1	0	96	
	piperacillin-tazobactam (TPZ)	28.1 (21–32)	0	0	97	
	ticarcillin-clavulanic acid (TTC)	28.6 (22–31)	0	1	96	
Cephalosporins	ceftazidime (CAZ)	28.1 (23–31)	0	0	97	
	cefuroxime (CXM)	24.9 (20–28)	0	97	0	
Carbapenems	imipenem (IMP)	21.3 (12–25)	9	34	54	
	meropenem (MEM)	32.0 (24–36)	0	0	97	
Monobactams	aztreonam (ATM)	30.8 (25–35)	2	0	95	
Fluoroquinolones	nalidixic acid (NA)^a^	36.4 (16–38)	0	4	93	
	ofloxacin (OFX)	30.8 (20–35)	3	3	91	
Aminoglycosides	kanamycin (K)^a^	22.5 (20–26)	0	0	97	
	streptomycin (S)^a^	14.9 (13–20)	0	38	59	
Macrolides, lincosamides and streptogramins	azithromycin (AZM)	24.4 (13–30)	0	0	97	
Tetracyclines	doxycycline (DO)^a^	19.9 (10–26)	5	2	90	
	tetracycline (TE)^a^	23.9 (0–28)	7	0	90	
Miscellaneous agents	chloramphenicol (C)	29.1 (22–34)	0	0	97	
	trimethoprim-sulfamethoxazole (TS)	32.0 (28–35)	0	0	97	

*R- Resistant, I- Intermediate sensitive, increased exposure, S-Susceptible, standard dosing regimen; **No.- numer of strains

^a^ interpretation of the CLSI breakpoints (CLSI Version 1.0, updated 15 May 2023)

**Table 2:** Multidrug resistance profiles and phylogenetic groups of *E. coli* isolates from urban pigeon faecal

Multidrug resistance profile	Phylogenetic group					Total No.** (%)
	A	B1	B2	D	X*	
IMP	–	–	–	5	–	5 (5.2)
TE	–	–	–	2	–	2 (2.1)
ATM	–	–	–	1	–	1 (1.0)
AMC	–	–	–	1	–	1 (1.0)
ATM, IMP	–	1	–	–	–	1 (1.0)
DO, TE	–	–	–	–	1	1 (1.0)
AMC, PRL, IMP	–	–	–	1	–	1 (1.0)
DO, TE, OFX	–	–	–	1	–	1 (1.0)
DO, TE, IMP	–	–	–	1	–	1 (1.0)
DO, TE, IMP, OFX	–	–	–	1	–	1 (1.0)
DO, TE, AMC, OFX	–	–	–	–	1	1 (1.0)
Total of resistant strains (%)	0 (0.00)	1 (1.0)	0 (0.00)	13 (13.4)	2 (2.1)	16 (16.5)
Total of non-resistant strains (%)	15 (15.5)	6 (6.2)	3 (3.1)	57 (58.8)	0 (0.00)	81 (83.5)
Total no. (%)	15 (15.5)	7 (7.2)	3 (3.1)	70 (72.2)	2 (2.1)	97 (100.00)

*X- undefined phylogenetic group; ** No.- numer of strains

**Fig. 2:**
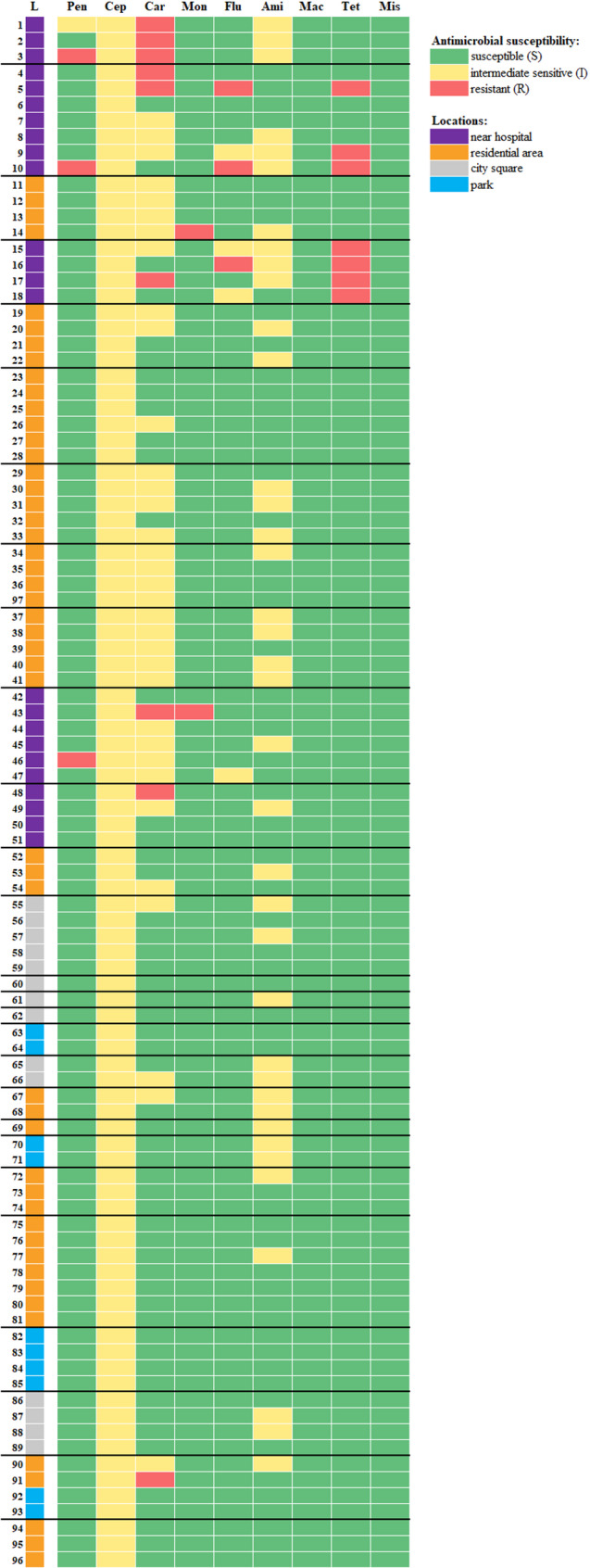
Heat map of phenotypic antibiotic resistance patterns in antibiotic classes of *E. coli* strains

The highest antibiotic resistance for different antibiotic classes was obtained in *E. coli* isolates (14/24, 58.3%) from areas near hospitals and in all these locations at least one strain was resistant (Fig. 2). A small percentage of resistant strains was found in pooled samples from residential areas (2/49, 4.1%). All *E. coli* strains from pooled samples originating from parks and urban squares were susceptible or intermediate sensitive in all antibiotic classes (Fig. 2).

The highest frequency of resistance strains was found in group D (13/97, 13.4%). The remaining resistant strains belonged to group B1 (1/97, 1.0%) and phylogenetic group not identified (2/97, 2.1%). Most *E. coli* strains showed resistance to one antibiotic (9/16, 56.3%).

The presence of genes related to the formation of the efflux pump AcrAB-TolC was found in 94.8% (92/97) of the isolates. The simultaneous absence of the *acrB* and *tolC* genes was detected in two *E. coli* isolates. In two isolates only the *acrB* gene was absent. The genes necessary to form this complex were not detected in only one isolate.

Green cells indicate the antibiotic class to which the *E. coli* strains showed a susceptibility profile, and yellow cells indicate intermediate susceptibility profiles. The resistant profile is indicated by a red cell. Antibiotic classes are abbreviated as follows: penicillins (Pen), cephalosporins (Cep), carbapenems (Car), monobactams (Mon), fluoroquinolones (Flu), aminoglycosides (Ami), macrolides, lincosamides, and streptogramins (Mac), tetracyclines (Tet), miscellaneous agents (Mis). The location of the collected samples (L) is indicated by coloured cells: purple - near the hospital, orange - residential area, gray - city square, blue - park. A horizontal black line separates *E. coli* strains isolated from the pooled sample from a specific location.

### Molecular confirmation of *E. coli* DNA

Molecular detection of the sequence of a variable fragment of the 16 S rRNA gene using three primers simultaneously (16E1 + 16E2 + 16E3) was confirmed that 97 of 100 strains were belonged to the *E. coli* species, and 21.7% (21/97) and 8.3% (8/97) were found to be “*pathogenic*” and “*non-pathogenic*”, respectively. In 63.9% (62/97) *E. coli* strains were not differentiated and the isolates contained both amplification products. A negative PCR result was obtained in 6.18% (6/97) of isolates, despite species confirmation.

Sequences of representative “*pathogenic*”, “*non-pathogenic*” and mixed *E. coli* strains were submitted to the GenBank database under accession numbers: PQ632963.1 to PQ632972.1.

### Phylogenetic group and virulence genes determination

Ninety-five of the 97 *E. coli* isolates were successfully assigned to one of the four main phylogenetic groups. The most common was group D (72.2%, 70/97), followed by group A (15.5%, 15/97), B1 (7.2%, 7/97) and B2 (3.1%, 3/97) (Table 3).

**Table 3:** Patterns of virulence genes identified in phylogroups of *E. coli* strains isolated from pigeon faeces

Virulence genes pattern	Phylogenetic group (No.*)					Total No. (%)
	A	B1	B2	D	X**	
*eaeA*	–	–	–	2	–	2 (2.06)
*stx*_*1*_	9	7	1	48	–	65 (67.01)
*stx*_*2*_	–	–	–	3	–	3 (3.09)
*stx*_*1*_ + *katP*	–	–	–	1	1	2 (2.06)
*eaeA* + *stx*_*1*_	6	–	–	4	–	10 (10.31)
*eaeA* + *stx*_*1*_ + *katP*	–	–	–	3	1	4 (4.12)
*eaeA* + *stx*_*1*_ + *katP* + *lpfA*_*O157/OI−154*_	–	–	–	1	–	1 (1.03)
*eaeA* + *stx*_*1*_ + *lpfA*_*O157/OI−141*_	–	–	–	4	–	4 (4.12)
*eaeA* + *stx*_*2*_ + *tir*	–	–	–	1	–	1 (1.03)
*eaeA* + *stx*_*2*_ + *tir* + *iha*	–	–	1	–	–	1 (1.03)
*eaeA* + *stx*_*2*_ + *tir* + *iha* + *katP*	–	–	1	–	–	1 (1.03)
*eaeA* + *katP*	–	–	–	3	–	3 (3.10)
Total of non-virulence isolates (%)	0 (0.00)	0 (0.00)	0 (0.00)	0 (0.00)	0 (0.00)	0 (0.00)
Total no. (%)	15 (15.46)	7 (7.22)	3 (3.09)	70 (72.17)	2 (2.06)	97 (100.00)

*No. – number of isolates; **X – undefined phylogenetic group

The frequency of the EPEC gene (*eaeA* only) was 5.2% (5/97), while for the EPEC and STEC genes (*eaeA* and *stx*_*1*_ and/or *stx*_*2*_) it was 22.7% (22/97). The *stx*_*2*_ gene, which is a more virulent variant of the *stx* gene encoding the Shiga toxin, was detected in a total of 6.2% (6/97) samples, only in phylogenetic groups D and B2 (4/6 and 2/6 isolates, respectively).

In 50.0% (3/6) of the isolates in which the *stx*_*2*_ gene was detected, the gene encoding the adhesion protein (intimin) and the translocated intimin receptor (*tir* gene) were also detected. In total, out of 17 virulence genes tested, 8 genes were detected, including *stx*_*1*_ (88.7%; (86/97), *eaeA* (27.8%; 27/97), *katP* (11.3%; 11/97), *stx*_*2*_ (6.2%; 6/97), *lpfA*_*O157/OI−141*_ (4.1%; 4/97), *tir* (3.1%; 3/97), *iha* (2.1%; 2/97) and *lpfA*_*O157/OI−154*_ (1.0%; 1/97). At least one of the virulence genes was found in all samples tested. More than one gene was present in 29.9% (29/97) of the *E. coli* isolates (Table 3).

## Discussion

Most epidemiological studies on wild birds focus on migration as a risk factor for the transmission of zoonotic pathogens to farms and the environment. Much data is available on the prevalence of diarrhoeal and MDR *E. coli* strains in migratory ducks, geese, quails, swans, cormorants, and other wild bird species [40–43]. The source of pathogenic *E. coli* may also be the faeces of synanthropic bird species, e.g., feral pigeons, which commonly and numerously inhabit urban agglomerations, however, data on the prevalence of different *E. coli* pathotypes are limited. Some studies have analysed samples from a wide range of host birds, including pigeons [20, 44, 45]. Most of them focus on detecting the *eaeA*, *stx*_*1*_ and *stx*_*2*_ genes as the genetic determinants of common EPEC and STEC strains, which are one of the main causes of diarrhoeal diseases in children and adults around the world. The prevalence of potentially diarrhoea-causing human *E. coli* strains in urban pigeon faeces was estimated at 9.4% in Iran [46], 10.7% in Rome [47], and 12.1% in Brazil [48]. In Brazil, Silva et al. [48] identified 7.1% enterotoxigenic and 0.5% enteroinvasive strains among all diarrheagenic *E. coli* detected. Atypical EPEC was also found in four samples from urban pigeons in Brazil [20] and three samples from pigeons in Hungary [49].

The average total number of *E. coli* obtained in the current study (8.78 × 10^7^ CFU/g) was lower than the results obtained by Karim et al. [50] in oral and cloacal swabs samples from individual birds 8.78 × 10^7^ CFU/g and 6.8 × 10^9^ CFU/ml, respectively. Karim et al. [50] and Sacristán et al. [51] found the presence of *E. coli* in 50.0% and 78.6% of cloacal swabs, respectively. While Silva et al. [48] and Ghanbarpour and Daneshdoost [46] found higher recoveries for fresh urban pigeon faeces, 78.6% and 89.6%, respectively. As shown by the above authors, the recovery of *E. coli* strains from individual bird samples is within the range estimated in this study for pooled faecal samples (25–100%). Differences in the total number of bacteria obtained by microbiological culture methods may vary depending on the location of the pigeon population under study, the source of biological material (e.g., cloacal swabs, faeces), or the type of medium used.

In the current study, most of the isolated strains belonged to phylogenetic groups related to ExPEC and diarrheagenic *E. coli*, including 72.2% to group D and 3.1% to group B2. The *eaeA* gene, which determines the production of intimin (adhesin) necessary for the attachment of bacteria to epithelial cells and typical of EPEC, as well as genes responsible for the production of Shiga toxins and other virulence factors, were identified in 18 and 15 strains, respectively (Table 3). Although the prevalence of the specific *E. coli* phylogenetic groups isolated in urban pigeons may vary [21, 46, 52], the *eaeA*, *stx*_*1*_ and *stx*_*2*_ genes appear to be common in these bird populations. In Europe, there are only a few reports on the occurrence and characteristics of the pathogenic *E. coli* (STEC and EPEC) originating from feral pigeons in cities. The frequency of these genes obtained in the current study (37.1% *eaeA*-positive, 88.7% *stx*_*1*_-positive and 6.2% *stx*_*2*_-positive) is higher than that reported in other studies. In Spain, 6% (2/33) *E. coli* isolated from urban pigeons were positive for *eaeA* gene and negative for *stx*_*1*_ and *stx*_*2*_. However, only *eaeA*-positive strains were analysed for the toxin [51]. The presented results showed that these genes can be detected independently of each other (Table 3). In research conducted in Finland, the *eaeA* gene was confirmed in two (6.9%) pigeons out of 29 tested [53]. Gargiulo et al. [54] showed the prevalence of *E. coli* O157 in 7.8% of cloacal swab samples collected from a total of 1,800 urban pigeons in Italy. The genes responsible for the production of Shiga toxin and encoding intimin in STEC and EPEC *E. coli* strains were isolated from faeces of urban pigeons in Germany [55], Portugal [56], Iran [46], Brazil [20].

In the current study, the virulence genes of translocated intimin receptor (*tir*) on the locus of enterocyte effacement pathogenicity island, gene encoding IrgA homologue adhesin (*iha*), two markers of long polar fimbriae (*lpfA*_*O157/OI−154*_, *lpfA*_*O157/OI−141*_) and catalase-peroxidase gene (*katP*) were detected in STEC strains and only in phylogenetic group D and B2. According to Kobayashi et al. [57], these genetic features include: those associated with high virulence of various STEC seropathotypes (A, B, C, D/E) and the course of the disease in humans. Bujňáková et al. [58] showed the prevalence of other virulence genes in a group of carrier and domestic pigeons in Slovakia, but similarly to this study, the largest number of them was obtained for *E. coli* strains from groups B2 and D. To the best of our knowledge, the results of the current work are among the first in Europe focusing on the occurrence of virulence factors other than commonly researched *eaeA*, *stx*_*1*_ and *stx*_*2*_ gene in feral pigeons from urban agglomeration. So far, only Borges et al. [20] investigated the presence of the *iha*, *saa*, *lpfA*_*O113*_, *espA*, *nlB* and *nlE* genes among *E. coli* isolates from free-living urban pigeons in Brazil. The genes mentioned were previously detected in clinical samples from humans symptomatically infected with *E. coli*.

Antimicrobial resistance phenotypes of cultured *E. coli* strains from pigeon faeces were confirmed, with a low percentage of resistant strains of 16.5%, of which over 7.0% were classified as MDR. Karim et al. [50] obtained much higher results, where 95.2% of isolated *E. coli* from 20 pigeons raised under household conditions were resistant to at least one to six antibiotics, and 23.8% were found to be MDR, with the highest resistance found to ampicillin, amoxicillin, erythromycin, and tetracycline (over 50% of isolates each). The obtained results may be related to treatment with different groups of antibiotics in domestic pigeons, the development of resistance to another antibiotic due to cross-resistance or co-selection. Although domestic animals seem to be more predisposed as a source of resistant bacteria, a relatively high prevalence of MDR *E. coli* isolated from wild mammals (wild boars (*Sus scrofa*), hedgehogs (*Erinaceus europaeus*), red fox (*Vulpes vulpes*)) and wild birds (e.g., common kestrels (*Falco tinnunculus*), white storks (*Ciconia Ciconia*), red kites (*Milvus milvus*), booted eagles (*Hieraaetus pennatus*), tawny owls (*Strix aluco*) has been found (71.9%), with mainly resistant to ampicillin (84.4%), trimethoprim–sulfamethoxazole (43.8%), tetracycline (39.1%), nalidixic acid (39.1%), and amoxicillin–clavulanic acid (33.6%) [59]. Among wild birds rescued in North-Western Italy, including birds of prey and synanthropic species, MDR *E. coli* was demonstrated in 13.2% of isolates, with the highest resistance rate observed for cephalexin (84.6%), amoxicillin-clavulanic acid (39.6%), and tetracycline (31.5%) [60]. Bueno et al. [21] obtained resistance to at least one antibiotic in all isolates from healthy free-living pigeons, and MDR was much higher and amounted to 63.0%. The greatest resistance was found in the aminoglycoside class, with the highest percentage of resistance found to streptomycin (88.0%). In turn, of the 203 *E. coli* isolates obtained from wild pigeons tested in the Czech Republic, drug resistance was detected in only 3 isolates (1.5%) [61].

The resistance results of *E. coli* strains obtained in this study differ from those reported by other authors. Unexpectedly, the highest number of imipenem-resistant strains was detected (9.3%). Importantly, the imipenem-resistant isolates originated from collection points located near hospitals (Fig. 2). Imipenem, a member of the carbapenem class, is a bactericidal beta-lactam antibiotic used in hospitals to treat severe infections in humans. Furthermore, it is particularly relevant in the context of the global problem of bacterial resistance to carbapenems and has been classified as “critically important for human medicine.” [62]. Sabença et al. [59] showed that all *E. coli* isolates from faecal samples from different wild birds and mammals in Portugal were susceptible to imipenem. Other studies conducted in wild animals showed varied resistance profiles of bacteria, with the most common resistance to ampicillin (71.5%) and tetracycline (63.6%), but susceptibility to imipenem was not tested [63]. Imipenem resistance of almost 7.0%, exhibiting five different MDR phenotypes, was found in a population of wild Canarian Egyptian vultures (*Neophron percnopterus majorensis*) endemic to the Canary Islands, Spain [64, 65]. Bueno et al. [21] showed high imipenem resistance in pigeon strains (almost 40%) and lower resistance to tetracyclines (approximately 25%), including three strains resistant to both antibiotics simultaneously. Ghanbarpour and Daneshdoost [46], although they showed an overall high level of resistance in *E. coli* isolated from pigeons, did not include imipenem in their study.

The available literature data on antibiotic resistance in wild animals, especially synanthropic species, are very limited, and the results obtained are variable. Differences in the results of antibiotic sensitivity tests in wildlife may be caused by factors such as the type region or habitat (e.g., agricultural, urban areas), source and level of environmental contamination, wild animal species’ feeding strategies, movement and migration, and the degree of synanthropization of the animal species tested [29, 66]. It is also possible that there are previously unknown acquired mechanisms of antibiotic resistance among commensal *E. coli* strains of wild animals [67].

The drug resistance patterns in *E. coli* strains isolated from pigeons may differ depending on the studied population. Ghanbarpour and Daneshdoost [46] found twenty different resistance testing patterns for only eight selected antimicrobials. In this study, the level of resistance obtained in *E. coli* strains isolated from the faeces of feral pigeons living near hospitals (58.3%) may indicate a relationship between the environment and the occurrence of antibiotic resistance in bacterial populations. The low number of resistant *E. coli* strains in the other locations suggests that birds may have had limited exposure to resistant bacteria strains or anthropogenic antimicrobials. Since feral pigeons are not treated with antibiotics, it is suggested that acquired resistance is related to their urban environment and feeding behaviour [21, 61]. However, studies confirm that the prevalence of MDR strains of *E. coli* from these birds is quite high and may be an important factor in the spread of antibiotic resistance in urban environment. In addition, the presence of the complete set of AcrAB-TolC complex genes in almost all isolated strains found in the current study indicates the effectiveness of removing many antimicrobial agents from bacterial cells, including antibiotics. The AcrAB − TolC efflux pump contributes to multidrug resistance in gram-negative bacteria, especially in *Enterobacteriaceae* [68]. It appears that the role of synanthropic pigeons in acquiring, maintaining, and spreading antibiotic-resistant bacteria in urban ecosystems is still not fully understood, and further research is necessary.

### Limitations of the study

Despite careful observations of the locations where wild pigeons occur, it cannot be ruled out that the collective sample included a faecal sample from more than one individual. Pooling samples may have reduced the specificity of the research methods used and thus influenced the interpretation of the results. Therefore, tests for antibiotic resistance and the presence of virulence genes included only *E. coli* strains whose isolated DNA was confirmed by genetic methods. Although biochemical methods allow for preliminary identification of isolated bacteria, they may have some limitations. In this study, three strains (3/100, 3.0%) initially identified as *E. coli* were excluded from further research procedures due to the lack of confirmation by genetic methods.

## Conclusion

The faeces of feral urban pigeons are a source of both “*pathogenic*” and “*non-pathogenic*” *E. coli* strains, with mixed strains predominating. Most strains were classified into phylogenetic groups D and A, related to human sources. Twelve patterns of virulence genes were identified among *E. coli* strains, with a great predominance of the single gene *stx*_*1*_ encoding Shiga toxin 1. For the first time in Europe, the presence of the virulence genes: *tir*, *iha*, *lpfA*_*O157/OI−154*_, *lpfA*_*O157/OI−141*_, *katP* was confirmed in STEC strains from phylogenetic groups D and B2 of *E. coli* isolated from pigeon faecal samples. The highest resistance was observed for imipenem (IMP), tetracycline (TE), and doxycycline (DO), and these antibiotics were also involved in most of the observed resistance patterns, including MDR. The obtained results justify the implementation of preventive measures in cities and the introduction of surveillance programs for synanthropic pigeon populations. This may help to protect both the urban environment and public health.

## Supplementary Information

Below is the link to the electronic supplementary material.

Supplementary Material 1

Supplementary Material 2

## Data Availability

All data generated or analysed during this study are included in this published article. The partial sequences are deposited in GenBank under the following accession numbers: PQ632963.1, PQ632964.1, PQ632965.1, PQ632966.1, PQ632967.1, PQ632968.1, PQ632969.1, PQ632970.1, PQ632971.1, PQ632972.1.

## Abbreviations

µg: Microgram
16S rRNA: 16 Svedberg ribosomal ribonucleic acid
AM: Ampicillin
AMC: Amoxicillin-clavulanic acid
Ami: Aminoglycosides
ATCC: American Type Culture Collection
ATM: Aztreonam
AZM: Azithromycin
bp: Base pair
C: Chloramphenicol
Car: Carbapenems
CAZ: Ceftazidime
CC: ChromID Coli
Cep: Cephalosporins
CFU/g: Colony-forming unit per gram
CXM: Cefuroxime
DNA: Deoxyribonucleic acid
DO: Doxycycline
E. coli: Escherichia coli
EHEC: Enterohemorrhagic
ENT: Ears, Nose, and Throat
EPEC: Enteropathogenic
ExPEC: Extraintestinal pathogenic Escherichia coli
Flu: Fluoroquinolones
I: Intermediate sensitive
IMP: Imipenem
InPEC/IPEC: Intestinal pathogenic Escherichia coli
K: Kanamycin
MDR: Multidrug-resistant
MEM: Meropenem
MH: Mueller-Hinton
Mon: Monobactams
NA: Nalidixic acid
No.: Number of strains/isolates
OFX: Ofloxacin
Pen: Penicillins
PRL: Piperacillin
R: Resistant
R + EB: Rebecca agar with EB Supplement
S: Streptomycin
S: Susceptible
SAM: Ampicillin-sulbactam
STEC: Shiga toxin-producing
T3SS: The type III secretion system
TE: Tetracycline
Tet: Tetracyclines
TPZ: Piperacillin-tazobactam
TS: Trimethoprim-sulfamethoxazole
TTC: Ticarcillin-clavulanic acid
